# Challenges in developing and implementing international best practice guidance for intermediate-risk variants in cancer susceptibility genes: *APC* c.3920T>A p.(Ile1307Lys) as an exemplar

**DOI:** 10.1136/jmg-2024-109900

**Published:** 2024-05-02

**Authors:** Terri Patricia McVeigh, Fiona Lalloo, Ian M Frayling, Andrew Latchford, Katie Snape, Miranda Durkie, Kevin J Monahan, Helen Hanson

**Affiliations:** 1 Cancer Genetics Unit, Royal Marsden NHS Foundation Trust, London, UK; 2 The Institute of Cancer Research, London, UK; 3 Clinical Genetics Service, Manchester Centre for Genomic Medicine, Central Manchester University Hospitals NHS Foundation Trust, Manchester, UK; 4 St Vincent's University Hospital, Dublin, Ireland; 5 St Mark's the National Bowel Hospital and Academic Institute, London, UK; 6 Institute of Medical Genetics, University Hospital of Wales, Cardiff, UK; 7 Clinical Genetics, St George's University Hospitals NHS Foundation Trust, London, UK; 8 Sheffield Diagnostic Genetics Service, Sheffield Children's NHS Foundation Trust, Sheffield, UK; 9 Imperial College London, London, UK; 10 Faculty of Health and Life Sciences, University of Exeter Medical School, Exeter, UK; 11 Peninsula Regional Genetics Service, Royal Devon and Exeter NHS Foundation Trust, Exeter, UK

**Keywords:** Genetic Counseling, Genetic Predisposition to Disease

The National Health Service (NHS) in the UK is a publicly funded entity, and decisions regarding allocation of scarce resources for provision of services, including related to genetic testing and cancer surveillance, are based on clinical and cost-effectiveness of proposed interventions, and are grounded by ethical considerations related to justice—allocation of resources based on clinical need; equity—fair provision of services across the population; beneficence and non-maleficence—provision of genetic testing/surveillance for clinical benefit, and minimisation of associated harm related to overinvestigation or overdiagnosis.^1^

NHS-funded cancer screening in the general population is guided by Wilson and Jungner criteria.^2^ These criteria, originally described in 1968, emphasise certain factors that should be considered in evaluating a screening test—related to the condition (natural history and severity), the test (sensitivity and specificity), the actionability of a positive finding (effective treatment), as well as acceptability and cost-effectiveness.^2^ Priority for cancer surveillance is typically given to those individuals at highest risk of disease—where numbers needed to screen or treat to prevent disease-associated mortality are low.^3^ NHS-funded constitutional (germline) genetic testing has traditionally been restricted, for the most part, to those individuals in whom the likelihood of identifying a causative clinically actionable constitutional pathogenic variant is at least 10%,^4^ although expansion of testing is already increasing as associated cost of testing falls and therapeutic implications expand.^5^ NHS-funded constitutional genetic testing is also focused, dependent on the patient phenotype, and limited to genes for which strong evidence regarding gene–disease association exists.^6 7^ Furthermore, analysis and reporting of variants are restricted to those associated with intermediate to high penetrance and where identification of the variant has clinical utility. For situations where there is evidence suggesting low penetrance (typically defined as an OR of <2^8^) associated with a particular genotype or class of variant, these variants are not analysed or reported in current NHS practice. For example, analysis of *CHEK2* is largely restricted to truncating variants, which are known to be associated with higher breast cancer risks,^9^ and testing of low-risk susceptibility alleles in genes such as *MITF, MC1R* or *EGFR* is not currently offered.

In other healthcare systems, thresholds at which cancer surveillance is offered may be lower than the threshold at which such surveillance is offered within the NHS. This, in part, explains international variability in the extent of genetic testing offered, eligibility criteria for genetic testing and guidelines for management of carriers of variants associated with incomplete penetrance in different cancer susceptibility genes. As a consequence, this can pose significant challenges when trying to define international best practice guidance. The International Society for Gastrointestinal Hereditary Tumours (InSiGHT) is a multidisciplinary expert group committed to enhancing care of patients with hereditary gastrointestinal cancer predisposition. This group has recently published a position statement regarding cancer risks associated with the recurrent variant NM_000038.6(*APC*): c.3920T>A, p.(Ile1307Lys), referred to as p.I1307K hereafter for brevity.^10^ *APC* p.I1307K has been a source of discussion internationally, given ethnicity-specific risks and variability in cancer surveillance recommendations between different jurisdictions.

The authors of the InSiGHT position statement deserve commendation for attempting to address existing variability in practice and provide a succinct and informed review of the latest data. International variability in clinical practice is almost inevitable given the specific population needs and access to intervention within each NHS or system. The UK Cancer Genetics Group (UKCGG), a constituent group of the British Society of Genomic Medicine, has issued a response to the InSiGHT position statement,^11^ incorporating health priorities, opportunity cost and equity of access within the UK NHS, mindful that adoption of these recommendations may conflict with the aims of the authors of to standardise clinical management of carriers of this variant.

Familial adenomatous polyposis (FAP) is caused by constitutional pathogenic variants in *APC*. These variants are usually inherited from an affected parent, but may arise de novo, and mosaicism is not uncommon.^12^ The hallmark of FAP is the development of large bowel polyps, but there is wide variation in the colonic phenotype.^13^ Extracolonic manifestations are common and may be clinically important features. The constitutional *APC* variant p.I1307K is not associated with clinical features of FAP but is associated with a moderately increased risk of colorectal cancer in individuals of Ashkenazi Jewish heritage.^10^ This should be considered a separate allelic predisposition. The colorectal cancer risks associated with this variant are limited to individuals of Ashkenazi Jewish heritage and confer a low or, at most, moderate risk, with an OR of 1.5–2 in this specific group.^10^ A high population prevalence, with 1 in 28 individuals of Ashkenazi Jewish ancestry carrying at least one copy of the variant allele, complicates things further. Homozygous carriers with wide phenotypic variability have been reported, but data are scant, such that the exact risks in such individuals are unclear.^10^ Carrier frequency in individuals of other ancestries is much less frequent, and associated risk uncertain.^10^ In line with this, the InSiGHT group recommends predictive testing in first-degree relatives of probands of Ashkenazi Jewish heritage as well as colorectal cancer surveillance of identified carriers (heterozygous or homozygous) every 5 years starting from 45 to 50 years if of Ashkenazi Jewish heritage, but not in individuals of other ethnicities.^10^

Recommendations for enhanced screening are based on family history as well as genotype. Current British Society of Gastroenterology (BSG)/Association of Coloproctology of Great Britain and Ireland/UKCGG guidelines recommend that individuals at high familial colorectal cancer risk (>6-fold) have 5-yearly colonoscopy from age 40 years, and those at moderate colorectal cancer risk (approximately 2-fold to 6-fold risk), a one-off colonoscopy at age 55 years,^14^ before entry to the National Bowel Cancer Screening Programme (NBCSP), predicated on the impact of colonoscopy on the mitigation of cancer risk in these populations. Individuals at lowpopulation risk (<2-fold risk) are offered screening within the NBCSP, which currently includes 2-year faecal immunochemical test (FIT) from age 60 years. The role of FIT-based surveillance in carriers of the *APC* p.I1307K variant has not been specifically evaluated. Enhanced colonoscopic surveillance for carriers of low to intermediate risk alleles is not typically recommended in the absence of a family history unless there is a clear and proven survival benefit. Given that the estimated risks associated with *APC* p.I1307K are less than twofold, recommending enhanced surveillance beyond that recommended to individuals at moderate risk based on family history is inconsistent with equivalent colonoscopic surveillance strategies. In the absence of specific data detailing outcomes of endoscopic surveillance in carriers of *APC* p.I1307K, the age at which advanced adenomas develop is unclear, and therefore the risks and benefit of such surveillance are difficult to establish.

Identification of *APC* p.I1307K alone is unlikely to fully explain the phenotype of an individual fulfilling clinical testing criteria for testing of genes associated with colorectal cancer and polyposis, as outlined in the NHS Genomic Testing Directory, which broadly recommends testing in young onset or strong familial cases; thus, the clinical utility of reporting this variant as an isolated finding is uncertain. Furthermore, standard guidelines for interpretation and classification of variants in *APC* cannot be applied to this variant,^15^ which has fuelled variability in reporting, with some labs classifying as ‘variant of uncertain significance’ and others as a ‘low-risk allele’.^16^ The position statement of the InSiGHT group advocates that this variant should be reported as ‘pathogenic, low penetrance’.^10^ However, after discussion with relevant key stakeholders, and considering that other similar low-risk alleles are not reported, the UKCGG has recommended that this variant is not considered or reported as part of NHS-funded diagnostic *APC* testing.

Identification of the *APC* p.I1307K variant in a family may arise through other channels (eg, through private testing/testing in another country). However, we do not recommend predictive testing for this variant in unaffected relatives, irrespective of the ethnicity of the proband, nor would we recommend colonoscopic surveillance unless otherwise warranted based on family history and in line with current BSG guidance. If the relative of a known carrier requires diagnostic *APC* testing in their own right, they should be counselled that the familial variant will not be reported by an NHS laboratory, even if present in their sample.

Given that data in an unselected population would be valuable to inform future guidance, we have also encouraged laboratories, where possible, to make data regarding variant identification available for submission to the National Disease Registration service, where possible, to facilitate review of outcomes in an unselected population in the future. We have also suggested that if screening is offered to pre-symptomatic carriers of this variant without a family history of colon cancer, that this should be within the context of a prospective research study. Ideally, prospectively collated data in an unselected patient group should be collected, to inform future evidence-based recommendations focused on improving outcomes and specific to population health needs.

## References

[1] NHS commissioning > how commissioning is changing. 2023. Available: https://www.england.nhs.uk/commissioning/how-commissioning-is-changing/

[2] Wilson JMG , Jungner G . Principles and Practice of Screening for Disease. Geneva: World Health Organization Geneva, 1968.

[3] Rembold CM . Number needed to screen: development of a statistic for disease screening. BMJ 1998;317:307–12. 10.1136/bmj.317.7154.307 9685274 PMC28622

[4] NICE (National Institute for Health and Care Excellence) . Familial breast cancer: classification, care and managing breast cancer and related risks in people with a family history of breast cancer. Clinical guideline [Cg164]. 2013. Available: https://www.nice.org.uk/guidance/cg164

[5] Barwell J , Snape K , Wedderburn S . The new genomic medicine service and implications for patients. Clin Med (Lond) 2019;19:273–7. 10.7861/clinmedicine.19-4-273 31308102 PMC6752257

[6] Martin AR , Williams E , Foulger RE , et al . Panelapp crowdsources expert knowledge to establish consensus diagnostic gene panels. Nat Genet 2019;51:1560–5. 10.1038/s41588-019-0528-2 31676867

[7] NHS England . National genomic test directory. 2024. Available: https://www.england.nhs.uk/publication/national-genomic-test-directories/

[8] Spurdle AB , Greville-Heygate S , Antoniou AC , et al . Towards controlled terminology for reporting germline cancer susceptibility variants: an ENIGMA report. J Med Genet 2019;56:347–57. 10.1136/jmedgenet-2018-105872 30962250

[9] Dorling L , Carvalho S , Allen J , et al . Breast cancer risk genes - association analysis in more than 113,000 women. N Engl J Med 2021;384:428–39. 10.1056/NEJMoa1913948 33471991 PMC7611105

[10] Valle L , Katz LH , Latchford A , et al . Position statement of the International society for gastrointestinal hereditary tumours (insight) on APC I1307K and cancer risk. J Med Genet 2023;60:1035–43. 10.1136/jmg-2022-108984 37076288 PMC10646901

[11] UKCGG response to position statement of the International society for gastrointestinal hereditary tumours (insight) on APC I1307K and cancer risk. 2023. 10.1136/jmg-2022-108984 PMC10646901 37076288

[12] Jansen AML , Goel A . Mosaicism in patients with colorectal cancer or polyposis syndromes: a systematic review. Clin Gastroenterol Hepatol 2020;18:1949–60. 10.1016/j.cgh.2020.02.049 32147591 PMC7725418

[13] Crabtree MD , Tomlinson IPM , Hodgson SV , et al . Explaining variation in familial adenomatous polyposis: relationship between genotype and phenotype and evidence for modifier genes. Gut 2002;51:420–3. 10.1136/gut.51.3.420 12171967 PMC1773342

[14] Monahan KJ , Bradshaw N , Dolwani S , et al . Guidelines for the management of hereditary colorectal cancer from the British society of Gastroenterology (BSG)/Association of Coloproctology of great Britain and Ireland (ACPGBI)/United kingdom cancer Genetics group (UKCGG). Gut 2020;69:411–44. 10.1136/gutjnl-2019-319915 31780574 PMC7034349

[15] Spier I , Yin X , Richardson M , et al . Gene-specific ACMG/AMP classification criteria for germline APC variants: recommendations from the clingen insight hereditary colorectal cancer/polyposis variant curation expert panel. Genet Med 2024;26:100992. 10.1016/j.gim.2023.100992 37800450 PMC10922469

[16] National Center for Biotechnology Information . Clinvar; [Vcv000000822.92], Nm_000038.6(APC):C.3920T>A (P.Ile1307Lys). 2024. Available: https://www.ncbi.nlm.nih.gov/clinvar/variation/VCV000000822.92

